# AI-Based Prediction of Capital Structure: Performance Comparison of ANN SVM and LR Models

**DOI:** 10.1155/2022/8334927

**Published:** 2022-09-19

**Authors:** Jesus Cuauhtemoc Tellez Gaytan, Karamath Ateeq, Aqila Rafiuddin, Haitham M. Alzoubi, Taher M. Ghazal, Tariq Ahamed Ahanger, Sunita Chaudhary, G. K. Viju

**Affiliations:** ^1^Business School, Tecnológico de Monterrey, Monterrey, Mexico; ^2^School of Information Technology, Skyline University College, Sharjah, UAE; ^3^Business School, under grant of FAIR Tecnológico de Monterrey, Monterrey, Mexico; ^4^School of Business, Skyline University College, Sharjah, UAE; ^5^Center for Cyber Security, Faculty of Information Science and Technology, Universiti Kebangsaan Malaysia (UKM), Bangi, Selangor, Malaysia, UAE; ^6^Department of Management Information Systems, College of Business Administration, Prince Sattam Bin Abdulaziz University, Al-Kharj, Saudi Arabia; ^7^Computer Science and Engineering, Marudhar Engineering College, Bikaner, Rajasthan, India; ^8^Post Graduate Studies, University of Garden City, Khartoum, Sudan

## Abstract

Capital structure is an integral part of the corporate finance that sources the funds to finance growth and operations. Managers always have to maintain value of the firm to be higher than the cost of capital in order to maximize the shareholders wealth. Empirical studies have used sources of finance like debt and equity as variables of capital structure. A choice between debt and equity finance analyzes the firm's ability to perform under the financially constrained environment to attain the sustainable growth. Therefore, it gives rise to a dire need to estimate the cost of capital precisely. We examined the capital structure of top ten market capitalization of the stock markets included in MSCI Emerging index with the use of artificial neural networks, support vector regression, and linear regression in forecasting methods. The capital structure is measured as the proportion of total debt over total equity (Tang et al., 1991). Other financial ratios such as profitability, liquidity, solvent, and turnover ratios were considered as drivers of the capital structure. Applying logistic and hyperbolic tangent activation functions, it was concluded that ANN has a great potential of replacing other traditional forecasting models with the nonstationary data. This research contributes with a new dimension for estimation through different activation functions. There is a possibility of ANN dominance as compared to the other models applied for predictability in financial markets.

## 1. Introduction

Artificial intelligent systems (AI) and machine learning (ML) have transformed the decision-making from human with an aim to improve quality of business and investments [1]. The dynamic environment is accelerated with creation of complexity in systems resulting in increased competition for which there is a need for a quick and better information decision system. The application of AI tools like artificial neural network (ANN) serves as best alternative to estimate the returns and build a positive vision, deliver more value, and radically improve the quality of business. There are many advances of AI in business in past few years, but implementation in accounting and finance is at a nascent stage with high need for further extensive machine learning techniques [2].

Over the past few years, a progressive revolution in technology took a root in creating artificial intelligence system. With the COVID-19, AI growth is accelerated and aligned towards reorganization of the industries. The concept and applications of AI is subject of interest for the business and academicians. Neural networks consist of multiple simple processors arranged in a communicative network, each of which is programmed to perform one identical, elementary processing task [3]. ANNs are considered to be universal functional approximators and provide a continuous function for a desired accuracy. The real-world systems are nonlinear, and traditional existing model are limited in expressing the relationship for the data series. Thus, ANNs perform nonlinear modeling without prior knowledge between input and output variable serving a more general and flexible model forecasting tool [4].

Neural networks are considered to be promising tools for forecasting financial time series. The factors such as selection of input variables, architecture of the network, and quantity of training data have an impact on accuracy of the model. ANNs models are highly multicomputational and interconnected units where in each input unit is allocated with sets of weights, bias adjustment, and transfer function to transforms the bias and sum of the weighted inputs to determine the output value from the input unit [5].

Classification, clustering, predicting, forecasting, and pattern recognition problems are the basic features of ANN [6]. AIS domain holds a vibrant research and inhibits an advanced role of leadership. Few studies on research on accounting firms reported an extensive use of AI tools in the integrated audit support systems [7]. Elbashir et al. [8] emphasized on identification of management accounting practices by business intelligence vendors. AI research studies have gained tremendous momentum due to the ability to find the suitable patterns to predict the future financial events more accurately that will assist in managerial decision-making.

### 1.1. Motivation of the Research

The main motivation of the research is due to the explosive growth in the ML and AI tools in the advancement of the finance literature. In the multiple studies performed based on ANN, it was concluded that artificial intelligence had outperformed economic, statistical methods, and other general forecasting models.

The large number of datasets used in the study assist in predictions through training algorithms with high dimension and increase in the number of variables as compared to the traditional structures.

The main purpose of using MSCI index from emerging markets is due to availability of many growth opportunities, free trade agreements, increased investments, and the capital flow. Nature of capital as prior to financial crisis was characterized by low debt levels, while after the crisis, higher debt levels are adopted. The rationale is to set a model in academic research, business managers, policy makers, and investors for real-time implementation and adjustment of capital structure based on predictions to maximize the firm's value [9].

Additionally, MSCI Emerging Markets Index (MSCI EMI) has accounted since 2000 for a Sharpe ratio of 0.39 and an annualized net return of 7.78%, which has outperformed the MSCI World and MSCI ACWI indexes. Within the MSCI EMI, China stands for a 35% of the country weights mean, while Brazil just 4% and Saudi Arabia less than 4%. Besides Brazil and Saudi Arabia show small weights within MSCI EMI, but in their index regions, they outstand with greater than 60% and 30%. So, despite country weights in one index, opportunities from companies of these countries cannot be misled as shown in the weights in other indexes [10].

This research addresses for a company's market capitalization criteria instead of an industry type selection. If all financial ratios were not available on any company, then it was selected the next lower market capitalization company. In that sense, the research intends to assess the capital structure forecasting capability of AI models of the highest market capitalization companies in emerging markets with no distinction of the industry type [11].

### 1.2. Main Objectives

This study contributes to application of artificial intelligence tools with an objective:

- To identify the best-fit model out of the three models namely ANN, SVR, and LR
- Predictive performance of the capital structure in the financial markets

## 2. Review of the Literature

Debt and equity are the two important variables of capital structure that provide the information to investors in the organization for supporting investment decisions. Research study by Pao [12] revealed the importance of debt with positive relationship with firm size and negative relationship with profitability, nondebt tax shield, and dividend. Large firms related with the more risk and more investment opportunity, so organizations need to develop the sustainable capital structure that maximize the firm growth and increase investment opportunity. A dynamic agency model was developed based on Lintner's adjustment model based on which managers choose to smooth dividends because they are trying to smooth their managerial rents through use of debt (leverage) and investment (capital expansion) to make sure that payouts are smoothed of [13, 14]. Similarly, another work by Hoang and Hoxha [15] used a sample of firms from 94 countries was explored with the countries in this study, Brazil, China, and Saudi Arabia. Payout policy is influenced by investment and debt policies and cannot be determined independently. In addition, it was revealed that geographic/cultural/institutional variation influence the response of payout policy to other corporate financing decisions. Another pioneer work was by Gatchev et al [16] that explored the interdependent and intertemporal nature of these decisions.

According to the research work [17–19] continuous audit, continuous monitoring with stream in the accounting field applies AI techniques. Artificial neural network (ANN) models drive uncertainty in finance through primarily involve in recognition of patterns in data and use these patterns to predict future events. ANNs predict currency movements and interest rate volatility for financial survival, thus claims to deal with the large noisy datasets.

According to Zahedi [20], ANNs translates the complexity of data into mathematical functions and provides analytical tools for business and economic systems that cannot be provided by traditional quantitative tools in statistics and econometrics.

Applications of ANN were classified into three categories like financial analysis prediction, classification of pattern, and optimization and control [21]. Sophisticated techniques employ time series model using ANN for forecasting future data points. Temporal effect determines the relationship of data patterns. Sequence of input data is considerable factor that depicts the relationship, and other types of ANN like recurrent and finite impulse response (FIR) are explored for dealing specific types of issues.

With the growing demand and transformation of the financial markets and the systems globally, the machine learning tools are extensively used, leading to extensive use of ANN technology in finance and accounting and literature for bankruptcy predictions, rating of the bond, forecasting in the stock markets, foreign exchange, evaluation of credit, and fraud detection [22]. Research work of Zhang & Maringer [23] used financial report data for items in the financial balance sheet for ratios such as return on equity, return on assets, price to earnings ratio, and debt to equity ratio.

The artificial neural network (ANN) technology identifies and reproduces a phenomenon or approximates a function without making the parameters explicit. It does not require any assumptions further like noisy, inconsistent, missing, and distribution of data. A wide and extensive literature is available on ANN applications. The high potential of ANN is identified through its highspeed processing model by academicians, researchers, and policy makers reflecting and considered as the superior performance of ANN compared to other econometric tools [24–28].

Four different technical indicators are used in the neural network architecture that include raw data, day of the week, prices of stocks are forecasted using the network that consists of input and output layer with the hidden layer between them [29, 30]. Back propagation of error is used to train the machine. Neural network topology is used for precise prediction. Its flexible characteristics make it universally applicable for financial analysis through technical indicators for the input data.

Recently, few studies have used artificial neural network (ANN) nonlinear models to resolve the forecasting problems [31] and focused on explaining capital structure by using time-series, cross-sectional tests, and panel data.

Financial economists predict increase in leverage with the fixed costs, nondebt tax shields, size of the firm, and investment opportunities [32]. According to research work of Moh'd et al. [33] employed time series data, and further results reveal that distribution of ownership equity is important in explaining overall capital structure, and managers try their best to reduce the level of debt to increase the owners wealth associated with the firm [34].

Extant literature is available on application of ANNs in classification and prediction problems in accounting and finance. ANN prediction in financial markets demonstrates the different techniques of the artificial intelligence tools that include technical and like fundamental analysis. A unique study on capital structure of G-7 countries except US related to factors similar to influence the capital structure of US firms by Rajan and Zingales [35] finds that leverage increases with asset structure and size but decreases with growth opportunities and profitability. ANN is based on certain biased functions where the machine is trained with simulated data to predict futuristic function by connecting with adaptive weights [36–38].

According to A. V. Devadoss, T. Ligori [39], financial markets are affected by a large number of external factors like economic and political in a complex way and making it difficult to predict precisely. It was supported by [40] that ANN provides the ultimate solution to this. ANN and SVM were used [41] to compare stock price index movements and conclude that ANN is better than SVM model.

On investigation of the best modeling of time series using Neural Network with four different approaches, i.e., raw data, raw data with a time index, detrending, and differencing for modeling, Qi and Zhang [42] concluded that Neural network gives better results.

Heo et al. [43] examined the descriptive and predictive power of ANN and OLS (Ordinary Least Squares) model to evaluate household's savings-to-income ratios and debt-to-asset ratios cross-sectional and across time. The results revealed ANN models serve as the best providers and serve as a better overall model fit to describe and forecast the financial ratios. The findings further indicate the importance of the machine learning procedures to provide a robust, efficient, and effective analytic method for lenders, financial service providers, academicians, and other researchers. Support Vector Machines (SVM) [45] and Artificial Neural Networks (ANN) are widely used for the prediction of stock price movements. Kuan and Liu (1995) investigated with empirical analysis on foreign exchange rates with recurrent and feed forward neural networks. According to Pao [12], analysis and forecasting of debt ratios achieved a greater accuracy using values of RMSE and ANN models as compared to other regression models [46]. Accordingly, it was suggested that ANN are capable of capturing varying nonlinear effects in the financial industries.

ANNs are more appropriate where the inputs are missing or nonlinear as compared to traditional statistical methods (Wang and Elhang, 2007). ANN model is a better choice for providing solutions involving complex practical problems because of their multidimensional analytical features as compared to other regression models. Factors such as number of observations, forecasting horizon, linear, or nonlinear form and data period effect the performance of ANN (Hill, O'Connor, and Remus [47]. According to the data period (annual, quarterly, monthly), functional form of the series (linear, nonlinear), forecasting horizon, and number of observations are the factors that affect the ANN performance. Based on the available literature, the ANN application in MSCI index for prediction of capital structure are least available. Most of the studies in this area have used a traditional regression model with one AI model either SVR or ANN. This study contributes with an objective to identify the best-fit model out of the three models namely ANN, SVR, and LR. Predictive performance of the capital structure is thereby assessed using the best model.

The paper is organized as follows: Section 2 presents review of the literature, Section 3 presents the data sources, variables, and methodology adopted. Sections 4 and 5 present the results of comparative study of the different models applied to rationalize the observed values.  Section 5 includes the summary and conclusions.

## 3. Data and Methodology

Dataset consists of 46 units of observations that comprise of 15 financial ratios taken from quarterly financial statements during the period Q1-2010 to Q2-2021.  Table 1 represents the details of the companies and the type of industry taken along with the market capitalization represented in their respective currencies. Nine companies belong to the top ten market capitalization within their stock markets and are listed in the MSCI Emerging Index. Equity index of Brazil, China and Saudi Arabia are taken from database of S&P Capital IQ.

Table 2 represents the details of the variables taken for the study of financial ratios to build our models. TDEQ represents the capital structure, and all the other ratios are taken with their respective formulae under description that can serve as reference to the readers.

(PetroChina) is China's biggest oil and gas producer and distributor and one of the largest oil and gas companies in the world. *C*hina Petroleum & Chemical Corporation is a vertically integrated energy and chemical company that is engaged in oil and gas exploration and production. China Tourism Group operates as a tourism company and provides tourism, hotel, scenic, passenger transport, and other travel services [50].

Saudi Telecom Company offers landline and fixed infrastructure, mobile and data services. Saudi electric company provides on the generation, transmission and distribution of electric power. Saudi Arabian Mining Company is responsible for all the mining activities.

Support vector regression and linear regression are widely used to predict. In our study, we give emphasis to build an ANN-based model.

ANNs are based on the principle of neuron connecting and sending signals with an attempt to simulate the learning process of the human brain by using complex algorithms. Every connection has a weight attached either with a positive or negative value associated. Positive weights activate the neuron while negative weights inhibit it. Generally setting up of ANN is six-step procedures. The data input the financial ratios used are defined and presented to the ANN as a pattern indicated with the desired outcome or target. Data are categorized as 80% of it as the training set or validation and 20% as the testing set from the sample data taken. The validation set used will assist in predictive ability and recommends when to stop the training of the ANN. As neural network consists of an input layer, an output layer, and one more intervening layers also known as hidden layers. These hidden layers capture the nonlinear relationship between variables. Each layer consists of multiple neurons that are connected to neuron in adjacent layers. With these neurons in multiple layers, these networks can capture complex phenomenon. This process of training set helps in computation of output with minimizing errors in forecast due to iterations. The algorithms used will adjust the model parameters connecting weights and nodes. The output of each processing unit is put forward through each layer if the network (Liu, Kuo and Sastri, 1995).

In this study, the main variable for analysis is Capital Structure (CS) measured as the proportion of Total Debt against Total Equity is considered to be as dependent variable. Independent variables are categorized as profitability, liquidity, solvency, and turnover ratios. These ratios are considered to be the drivers of capital structure (15 ratios are taken).

**Figure 1:**
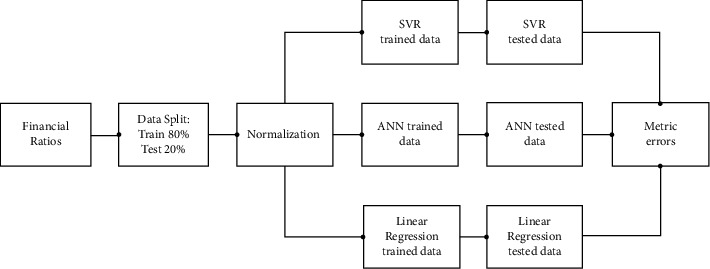
Methodology.

Figure 1 represents the methodology and describes the entire approach followed to assess the three models. The data are split into 80% and 20% as stated before and are normalized. After the normalization, the data are fed into three models: SVR, LR, and ANN. After the models are trained on the 80% data, remaining 20% data are used to evaluate the models The root mean squared error (RMSE) and mean absolute error (MAE) are the metric errors approach to compare performance among models. Estimations were performed using *R* version 4.0.5.

The following section is used to set a detailed context on normalization, error metrics using ANN model as an example.

The normalization step helps to make sure that large values do not overcome features with small values. In that sense, the original financial ratio matrix is transformed to a matrix of range (0, 1). The following expression represents the normalized value:

(1) $$\begin{array}{c} x_{norm}=\frac{x-x_{\text{min}}}{x_{\text{max}}-x_{\text{min}}}. \end{array}$$

After normalization, data are split into training (80%) and testing (20%) data. Different ANN structures were estimated based on number of hidden layers and number of nodes. The Logistic (sigmoid) and Hyperbolic Tangent (tanh) activation functions were applied as transfer functions from the weighted-sum inputs to determine the output.

**Figure 2:**
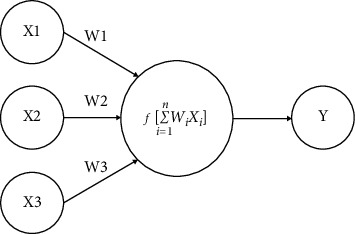
ANN structure.

Figure 2 represents a general model of an ANN structure with 3 inputs (X1, X2, and X3), one hidden layer, one node, and the output (Y); where *f* represents the activation function.

The following expression shows the logistic function:

(2) $$\begin{array}{c} \varphi \left(x\right)=\frac{1}{1+e^{-x}}. \end{array}$$

However, the tanh function is shown in the following expression:

(3) $$\begin{array}{c} \tanh\left(x\right)=\frac{e^{2x}-1}{e^{2x}+1}. \end{array}$$

The performance comparison among algorithms is analyzed through the root mean squared error (RMSE) and mean absolute error (MAE) metrics. Expression (4) shows the RMSE metric.

(4) $$\begin{array}{c} RMSE=\sqrt{\frac{1}{n}\overset{n}{\underset{i=1}{\sum }}{\left(x_{i}-{\bar{x}}_{i}\right)}^{2}}. \end{array}$$

And, the following expression shows the MAE metric:

(5) $$\begin{array}{c} MAE=\sqrt{\frac{1}{n}\overset{n}{\underset{i=1}{\sum }}\left|x_{i}-\bar{x_{i}}\right|}. \end{array}$$

## 4. Comparison of Models

The results obtained using different models are compared in this section using the error metrics defined in the previous section. The three models are now predicted for different regions and their RMSE, RSE, RAE, and MAE are evaluated. For ANN models, different combinations of layers and nodes are experimented along two functions. Hence, the discussion of results is made on two folds, first to discuss on the best suitable structure of ANN model and then compare it with rest of the two models.

### 4.1. Brazil

Tables 3–5 reveal the comparison of different applications and the metric errors of the three algorithm approaches used in forecasting the capital structure. The ANN results are based on the best algorithm structure with output that had the least RMSE (refer Table 6).

**Figure 3:**
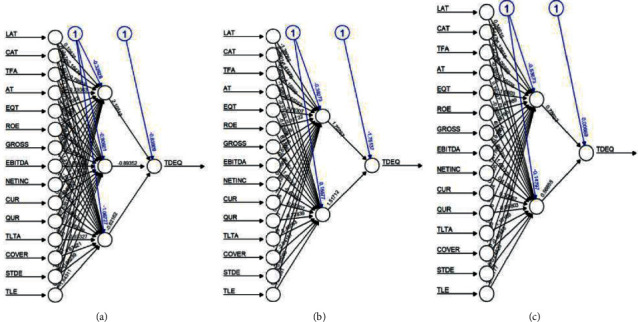
ANN structures.

As per the ANN structures shown in Figure 3 for the Brazilian companies taken from equity index BOVESPA Vale, Petroleo Brasileiro, and Weg are shown with neural network structures.

As per algorithm, SVR has outperformed in Weg with RMSE value of 0.0464, while Vale and Petroleo Braileiro had LR with 0.0610 and LR of 0.0486, respectively.

### 4.2. China

**Figure 4:**
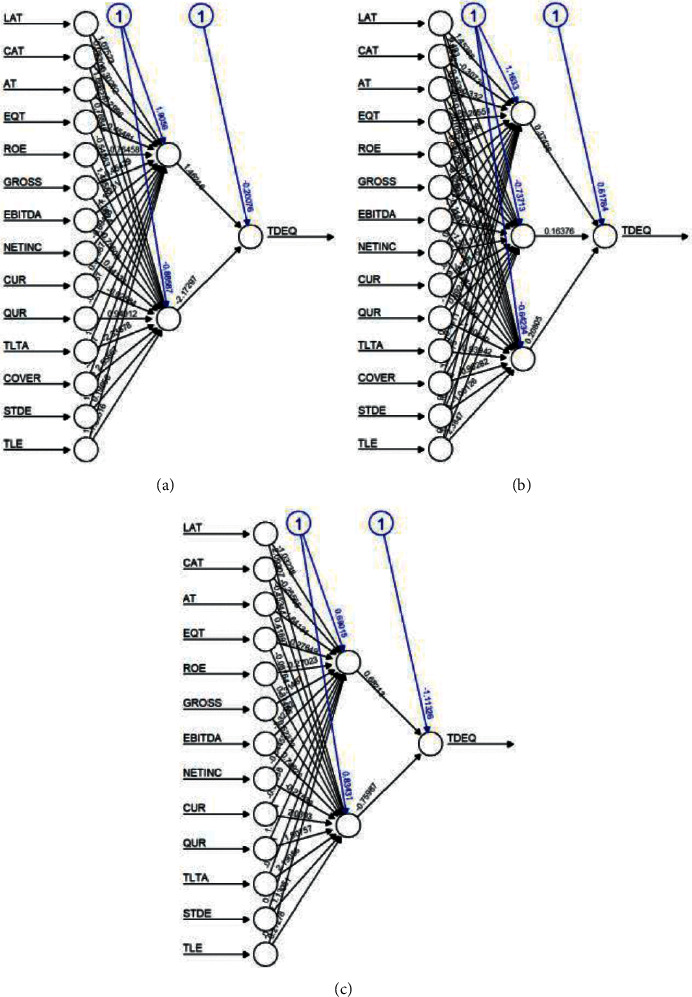
ANN structures of China. (a) Petro China. (b) Petro Chemical. (c) Tourism.

With reference to Figure 4 the algorithmic metrics are validated; in Petro China ANN performed better with 0.0426 and SVR and LR were similar with 0.0759 and LR with 0.0758. China petroleum and chemical works well with LR with 0.0708 as its error. SVR (0.1117) outperformed against the ANN and LR in China Tourism.

Based on the ANN structures through activation functions of logistics and tanh for all the layers and all nodes, the best model fit is tanh function indicates that ANN can predict better in nonlinear values.

### 4.3. Saudi Arabia

**Figure 5:**
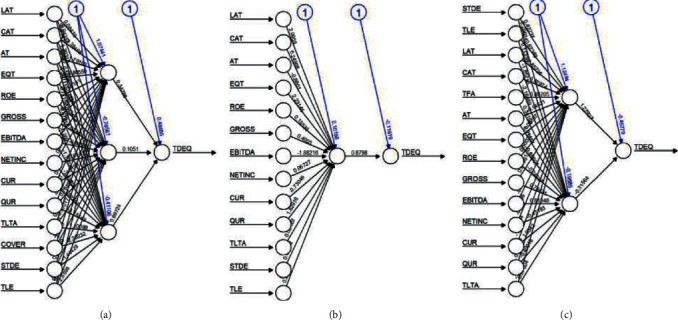
ANN structures of Saudi Arabia. (a) Telecom. (b) Electricity. (c) Mining.

The ANN structures algorithm metrics indicate 0.0328 for LR in Saudi Telecom as per RMSE followed by ANN and SVR with 0.0807 and 0.1274, respectively, as reflected in Figure 5. In Saudi Electricity, SVR 0.0618 is considered as best-fit followed by ANN with 01198 and LR with 0.1267. For Saudi Arabian Mining sector, LR model was best with 0.0283 compared to 0.0678 and 0.0705 for ANN and SVR, respectively.

Based on the ANN structures through activation functions of logistics and tanh for all the layers and all nodes, the best model fit indicates ANN can predict better in nonlinear values.

## 5. Results and Discussion

Empirical results of ANN structures are validated in the tables in terms of Algorithm metric errors using recognized statistical tools RMSE and MAE. Results indicate the capital structure prediction performance based on sole ANN structure. All ANN structures fitted well as one layer-one node structure with the same activation function and assists to transfer the inputs combination to the capital structure forecast output, thus considering ANN as an ideal promoting tool.

Equity index of Brazil, such as Vale, Petroleo, and Weg, were well fitted by the tanh function, which revealed best ANN network performance than logistic function.

When activation functions were applied for industry wise comparison in oil and gas companies of China, both logistic and tanh function performed well indicating that ANN is an ideal model for predictive performance.

Capital structure pattern of all three oil and gas companies, the Petroleo Brasileiro (Brazil) and Petroleum Chemical (China) and Petro China showed an average capital structure ratio, Petroleo Brasileiro and Petrochemical China were fitted by the logistic and tanh functions, respectively. The Saudi Arabian companies showed the best ANN structure based on the same activation function. Their average capital structures ratios were 24.55% (Telecom), 16.2% (Electricity), and 21.63% (Mining). It is concluded that when companies taken for the study are from same country then ideally same activation function could be fitted irrespective of their size of capital structure.

In Brazil and China, the sample consists of other industry group hence same activation function will not be applicable. The comparative analysis applied among the ANN estimated structures revealed that common features as those of country, industry, or capital structure level that could justify a sole activation function in forecasting the capital structure of companies belonging to emerging stock markets. Thus, based on the research work, it can be concluded that for the nonlinear data, ANN models are considered to be the best as compared to the other models applied in the study. In the sample taken for the study, LR models were identified as the best model for the linear data. When the data are nonlinear, ANN predictions were the most suitable models. Hence, common features as those of country, industry, or capital structure level may also indicate similar results.

## 6. Conclusions

With the application of algorithm, ANN model is considered to be the best-fit model in predictive performance as revealed and validate through low RMSE. The activation function of both logistic and tanh indicates low RMSE and support ANN structures. LR and SVR outperformed for the linear data. This research work supports the earlier studies that consider ANN network as superior in comparison with another model.

This study emphasized on the prediction of capital structure through input variables as financial ratios and algorithm error metrics applied in ANN, SVR, and LR models. The study was limited for predictive performance only. Although financial ratios were used as inputs, the relationship among the ratios is not measured and is not included. Authors plan to extend this work to evaluate and predict the capital structure in future research work with individual applications of ratios in terms of profitability, liquidity, solvency, and turnover ratios .

## Figures and Tables

**Table 1:** Data representing the stock market indices of different companies with industries and market capitalization representing local currency.

Equity index	Company	Industry	Market capitalization (millions of the trading currency as of July 2021)
BOVESPA (Brazil)	Vale	Materials (steel)	565,010.2 BRL
	Petroleo Brasileiro	Energy (integrated oil and gas)	376,650.0 BRL
	Weg	Industrials (electrical components and equipment)	156,088.6 BRL

SSEC (China)	PetroChina Company	Energy (integrated oil and gas)	970,735.0 HKD
	China Petroleum and Chemical Corp.	Energy (integrated oi and gas)	592, 658.1 HKD
	China Tourism Group	Consumer discretionary	478,629.0 HKD

Tadawul (Saudi Arabia)	Saudi Telecom Company	Communication services (integrated telecommunication services)	271,194.9 SAR
	Saudi Electricity Company	Utilities (electric utilities)	112,289.7 SAR
	Saudi Arabian Mining Company	Materials (diversified metals and mining	85,895.3 SAR

*Note*.: Vale is the world's largest producer of iron ore, pellets, and nickel in Brazil. Petroleo Brasileiro is an oil and gas company also known as Petrobras. WEG  is a Brazilian company, operating worldwide in the electric engineering, power, and automation technology areas [48, 49]. BRL: Brazilian Real, HKD: Hong Kong Dollar, and SAR: Saudi Riyal.

**Table 2:** Variables.

Variables	Ratio	Description
TDEQ (main)	Capital structure	Total debt/equity
LAT	Liquid asset turnover	Revenues/cash and equivalents
CAT	Current asset turnover	Revenues/current assets
TFA	Tangible fixed assets	Revenues/tangible fixed assets
AT	Asset turnover	Revenues/assets
EQT	Equity turnover	Revenues/equity
ROE	Profitability	Net income/equity
GROSS	Profitability	Gross margin
EBITDA	Profitability	Ebitda margin
NETINC	Profitability	Net income margin
CUR	Liquidity	Current assets/current liabilities
QUR	Liquidity	(Cash and short investments)/current liabilities
STDE	Liquidity	Current liabilities/equity
TLTA	Solvency	Total liabilities/total assets
COVER	Solvency	EBIT/interest expenses
TLE	Solvency	Total liabilities/equity

**Table 3:** Algorithm metric errors for Brazil.

Country	Company	Metric	Model		
			ANN	SVR	LR
BRAZIL	Vale	RMSE	0.1008	0.0800	0.0610
		MAE	0.0825	0.0617	0.0503
	Petroleo Brasileiro	RMSE	0.0805	0.1314	0.0486
		MAE	0.0581	0.0771	0.0431
	Weg	RMSE	0.0685	0.0464	0.0541
		MAE	0.0542	0.0393	0.0411

**Table 4:** Algorithm metric errors.

Country	Company	Metric	Model		
			ANN	SVR	LR
CHINA	PetroChina	RMSE	0.0426 ^*∗*^	0.0759	0.0758
		MAE	0.0307 ^*∗*^	0.0580	0.0540
	China Petroleum and Chemical	RMSE	0.1094	0.1026	0.0708
		MAE	0.0945	0.0849	0.0533
	China Tourism	RMSE	0.1562	0.1117	0.1371
		MAE	0.1414	0.0727	0.1125

^∗^statistical significance at 0.05 percent level.

**Table 5:** Algorithm metric errors.

Company	Metric	Model		
		ANN	SVR	LR
Saudi Telecom	RMSE	0.0807	0.1274	0.0328
	MAE	0.0675	0.0879	0.0286

Saudi Electricity	RMSE	0.1198	0.0618	0.1267
	MAE	0.1008	0.0568	0.0994

Saudi Arabian Mining	RMSE	0.0678	0.0705	0.0283
	MAE	0.0583	0.0461	0.0168

**Table 6:** ANN structures performance based on metric errors.

Actfct	Layers	Nodes	RMSE	RSE	RAE	MAE
*Vale*						
Logistic	3	5,3,2	0.241	0.9947	0.9962	0.1887
	2	3,2	0.2417	1.0002	1.0063	0.1906
	1	3	0.1008	0.1742	0.436	0.0825
	1	2	0.1267	0.2255	0.4147	0.0968
	1	1	0.131	0.2937	0.5903	0.1118

Tanh	3	5,3,2	0.1415	0.3426	0.6277	0.1189
	2	3,2	0.1056	0.1911	0.5052	0.0957
	1	3	0.1557	0.4152	0.5678	0.1075
	1	2	0.1634	0.4573	0.7005	0.1326
	1	1	0.1048	0.1881	0.4664	0.0883

*Petroleo*						
Logistic	3	5,3,2	0.2668	0.9989	0.9995	0.2332
	2	3,2	0.2677	1.006	0.9998	0.2333
	1	3	0.0903	0.1144	0.2888	0.0674
	1	2	0.0805	0.0909	0.2491	0.0581
	1	1	0.1186	0.1974	0.3444	0.0803

Tanh	3	5,3,2	0.1436	0.2894	0.3899	0.091
	2	3,2	0.1091	0.1672	0.3689	0.0861
	1	3	0.0916	0.1179	0.3248	0.0758
	1	2	0.1177	0.1944	0.4191	0.0978
	1	1	0.2671	1.0016	1.0000	0.2334

*WEG*						
Logistic	3	5,3,2	0.2263	1.0124	1.0682	0.1667
	2	3,2	0.2261	1.0102	1.087	0.1696
	1	3	0.0914	0.1653	0.5598	0.0873
	1	2	0.0862	0.147	0.5071	0.0791
	1	1	0.2222	0.9762	1.0077	0.1572

Tanh	3	5,3,2	0.1067	0.2253	0.5387	0.084
	2	3,2	0.1262	0.3149	0.7485	0.1168
	1	3	0.1303	0.3356	0.7323	0.1143
	1	2	0.0685	0.0927	0.3477	0.0542
	1	1	0.2259	1.0085	0.10533	0.1644

*PETROCHINA*						
Logistic	3	5,3,2	0.2050	1.1133	1.1216	0.1793
	2	3,2	0.2017	1.0781	1.0990	0.1757
	1	3	0.0487	0.0628	0.2305	0.0368
	1	2	0.0426	0.0482	0.1922	0.0307
	1	1	0.0741	0.1456	0.3935	0.0629

Tanh	3	5,3,2	0.0924	0.2262	0.4569	0.0730
	2	3,2	0.1122	0.3335	0.5331	0.0852
	1	3	0.0753	0.1501	0.3914	0.0626
	1	2	0.0521	0.0719	0.2432	0.0389
	1	1	0.1387	0.5093	0.7344	0.1174

*Petrochem*						
Logistic	3	5,3,2	0.3361	1.0009	0.9967	0.2985
	2	3,2	0.1534	0.2085	0.4513	0.1352
	1	3	0.1131	0.1133	0.3241	0.0971
	1	2	0.1222	0.1323	0.3415	0.1023
	1	1	0.1595	0.2255	0.4945	0.1481

Tanh	3	5,3,2	0.1322	0.1547	0.3768	0.1128
	2	3,2	0.1516	0.2036	0.4244	0.1271
	1	3	0.1094	0.1061	0.3154	0.0945
	1	2	0.107	0.1014	0.2084	0.0894
	1	1	0.1969	0.3437	0.5739	0.1719

*Tourism*						
Logistic	3	5,3,2	0.1519	1.0218	1.0704	0.1295
	2	3,2	0.1555	1.0722	1.1574	0.1400
	1	3	0.1513	1.0138	1.0737	0.1299
	1	2	0.1562	1.0817	1.1687	0.1414
	1	1	0.1521	1.0251	1.1043	0.1336

Tanh	3	5,3,2	0.1410	0.8814	0.6781	0.0820
	2	3,2	0.1624	1.1693	1.2364	0.1496
	1	3	0.2540	2.8589	1.4560	0.1761
	1	2	0.2268	2.2797	1.1462	0.1387
	1	1	0.1938	1.6641	1.2561	0.1519

*Telecom*						
Logistic	3	5,3,2	0.3497	1.0025	1.0544	0.2999
	2	3,2	0.3477	0.9909	1.0505	0.2989
	1	3	0.0931	0.0710	0.2868	0.0816
	1	2	0.1479	0.1795	0.3433	0.0977
	1	1	0.1768	0.2562	0.3967	0.1129

Tanh	3	5,3,2	0.1593	0.2079	0.3160	0.0899
	2	3,2	0.0875	0.0627	0.2469	0.0702
	1	3	0.1263	0.1308	0.2958	0.0841
	1	2	0.0807	0.0534	0.2372	0.0675
	1	1	0.1113	0.1015	0.3275	0.0932

*Electricity*						
Logistic	3	5,3,2	0.2868	1.2767	1.0451	0.2274
	2	3,2	0.2837	1.2489	1.0613	0.2309
	1	3	0.2942	1.3437	1.0451	0.2274
	1	2	0.1341	0.2791	0.5437	0.1183
	1	1	0.2904	1.3087	1.0728	0.2334

Tanh	3	5,3,2	0.1356	0.2852	0.5227	0.1137
	2	3,2	0.2554	1.0128	0.8580	0.1867
	1	3	0.1785	0.4944	0.5931	0.129
	1	2	0.1357	0.2857	0.5387	0.1172
	1	1	0.1198	0.2227	0.4634	0.1008

*Mining*						
Logistic	3	5,3,2	0.2860	1.0046	1.0508	0.2218
	2	3,2	0.1315	0.2124	0.5990	0.1264
	1	3	0.1473	0.2665	0.6646	0.1403
	1	2	0.1293	0.2052	0.5718	0.1207
	1	1	0.1714	0.3608	0.7047	0.1487

Tanh	3	5,3,2	0.1280	0.2013	0.5327	0.1124
	2	3,2	0.1513	0.2811	0.6034	0.1274
	1	3	0.0678	0.0565	0.2757	0.0582
	1	2	0.1253	0.1927	0.5461	0.1153
	1	1	0.1737	0.3705	0.6685	0.1411

Note: Act.fct represents a differentiable function applied for smoothing the result of cross product of the covariate of neurons and the weights.

## Data Availability

Dataset consist of 46 units of observations that comprise 15 financial ratios taken from quarterly financial statements during the period Q1-2010 to Q2-2021 for the research, and they will be provided upon request.

## References

[1] Medsker L. (1996). Microcomputer applications of hybrid intelligent systems. *Journal of Network and Computer Applications*.

[2] Arya P., Bhagat A., Nair R. (2019). Improved performance of machine learning algorithms via ensemble learning methods of sentiment analysis. *International Journal on Emerging Technologies*.

[3] Vellido A., Lisboa P., Vaughan J. (1999). Neural networks in business: a survey of applications (1992–1998). *Expert Systems with Applications*.

[4] Hoang E., Hoxha I. (2021). A tale of two emerging market economies: evidence from payout smoothing in China and Taiwan. *International Journal of Managerial Finance*.

[5] Wong B., Lai V., Lam J. (2000). A bibliography of neural network business applications research:1994-1998. *Computers & Operations Research*.

[6] Nair R., Bhagat A. (2020). *An Introduction to Clustering Algorithms in Big Data*.

[7] Dowling C., Leech S. (2007). Audit support systems and decision aids: current practice and opportunities for future research. *International Journal of Accounting Information Systems*.

[8] Elbashir M., Collier P., Sutton S. (2011). The role of organizational absorptive capacity in strategic use of business intelligence to support integrated management control systems. *The Accounting Review*.

[9] Kara Y., Acar Boyacioglu M., Baykan O. K. (2011). Predicting direction of stock Price index movement using artificial neural networks and support vector machines: the sample of the Istanbul stock exchange. *Expert Systems with Applications*.

[10] Hoang E., Hoxha I. (2016). Corporate payout smoothing: a variance decomposition approach. *Journal of Empirical Finance*.

[11] Hoang E., Hoxha I. (2015). The sensitivity of payouts to corporate financing decisions. *Journal of Financial Economic Policy*.

[12] Pao H. T. (2008). A comparison of neural network and multiple regression analysis in modeling capital structure. *Expert Systems with Applications*.

[13] Lambrecht B., Myers S. (2012). A Lintner model of payout and managerial rents. *The Journal of Finance*.

[14] Lambrecht B., Myers S. (2017). The dynamics of investment, payout and debt. *The Review of Financial Studies*.

[15] Hoang E., Hoxha I. (2019). An international study of the response of corporate payout policy. *International Journal of Managerial Finance*.

[16] Gatchev V., Pulvino T., Tarhan V. (2010). The interdependent and intertemporal nature of financial decisions: an application to cash flow sensitivities. *The Journal of Finance*.

[17] Kuhn J., Sutton S. (2010). Continuous auditing in ERP system environments: the current state and future directions. *Journal of Information Systems*.

[18] Brown-Liburd H., Issa H., Lombardi D. (2015). Behavioral implications of big data’s impact on audit judgment and decision making and future research directions. *Accounting Horizons*.

[19] Vasarhelyi M., Kogan A., Tuttle B. (2015). Big data in accounting: an overview. *Accounting Horizons*.

[20] Zahedi F. (1993). *Intelligent Systems for Business: Expert Systems with Neural Networks*.

[21] Rumelhart D. E., Widrow B., Lehr M. (1994). The basic ideas in neural networks. *Communications of the ACM*.

[22] Krishnaswamy C., Gilbert E., Pashley M. (2000). *Neural Network Applications in Finance: A Practical Introduction*.

[23] Zhang J., Maringer D. (2016). Using a genetic algorithm to improve recurrent reinforcement learning for equity trading. *Computational Economics*.

[24] Cheng B., Titterington D. (1994). Neural networks: a review from a statistical perspective. *Statistical Science*.

[25] Haykin S. (1999). *Neural Networks*.

[26] Hill T., Marquez L., O’Connor M., Remus W. (1994). Artificial neural network models for forecasting and decision making. *International Journal of Forecasting*.

[27] Zhang G., Eddy Patuwo B., Hu M. (1998). Forecasting with artificial neural networks. *International Journal of Forecasting*.

[28] Shamout M., Ben-Abdallah R., Alshurideh M., Alzoubi H., Kurdi B., Hamadneh S. (2022). A conceptual model for the adoption of autonomous robots in supply chain and logistics industry. *Uncertain Supply Chain Management*.

[29] Wong B., Selvi Y. (1998). Neural network applications in finance: a review and analysis of literature (1990–1996). *Information & Management*.

[30] Wong B., Bodnovich T., Selvi Y. (1997). Neural network applications in business: a review and analysis of the literature (1988–1995). *Decision Support Systems*.

[31] Altun H., Bilgil A., Fidan B. C. (2007). Treatment of multi-dimensional data to enhance neural network estimators in regression problems. *Expert Systems with Applications*.

[32] Yildiz B., Yalama A., Coskun M. (2008). Forecasting the Istanbul stock exchange national 100 index using an artificial neural network. *An International Journal of Science, Engineering and Technology*.

[33] Mohd M. A., Perry L. G., Rimbey J. N. (1998). The impact of ownership structure on corporate debt policy: a time–series cross–sectional analysis. *Financial Review*.

[34] Zhang G., Patuwo B., Hu M. (2001). A simulation study of artificial neural networks for nonlinear time-series forecasting. *Computers & Operations Research*.

[35] Rajan R. G., Zingales L. (1995). What do we know about capital structure? some evidence from international data. *The Journal of Finance*.

[36] Shadbolt J. (2002). *Neural Networks and the Financial Markets: Bpredicting, Combining, and Portfolio Optimisation*.

[37] Trippi R., DeSieno D. (1992). Trading equity index futures with a neural network. *The Journal of Portfolio Management*.

[38] Walczak S. (2001). An empirical analysis of data requirements for financial forecasting with neural networks. *Journal of Management Information Systems*.

[39] Devadoss A., Ligori T. (2013). Stock prediction using artificial neural networks. *International Journal of Data Mining Techniques and Applications*.

[40] Samek D., Varachha P. (2013). Time series prediction using artificial neural networks. *International Journal of Mathematical Models and Methods in Applied Sciences*.

[41] Ariyo Adebiyi, Ayo C., Adebiyi M. O., Otokiti O. (2012). Stock Price prediction using neural network with hybridized market indicators. *Journal of Emerging Trends in Computing and Information Sciences*.

[42] Min Q., Peter Zhang G. (2008). Trend time–series modeling and forecasting with neural networks. *IEEE Transactions on Neural Networks*.

[43] Heo W., Lee J., Park N., Grable J. (2020). Using Artificial Neural Network techniques to improve the description and prediction of household financial ratios. *Journal of Behavioral and Experimental Finance*.

[44] Cortes C., Vapnik V. (1995). Support-vector networks. *Machine Learning*.

[45] Kuan C. M., Liu T. (1995). Forecasting exchange rates using feedforward and recurrent neural networks. *Journal of Applied Econometrics*.

[46] Ramakrishna Y., Alzoubi H. (2022). Empirical investigation of mediating role of six sigma approach in rationalizing the COQ in service organizations. *Operations and Supply Chain Management: An International Journal*.

[47] Hill T., O’Connor M., Remus W. (1996). Neural network models for time series forecasts. *Management Science*.

[48] Lee K., Azmi N., Hanaysha J., Alzoubi H., Alshurideh M. (2022a). The effect of digital supply chain on organizational performance: an empirical study in Malaysia manufacturing industry. *Uncertain Supply Chain Management*.

[49] Lee K., Romzi P., Hanaysha J., Alzoubi H., Alshurideh M. (2022b). Investigating the impact of benefits and challenges of IOT adoption on supply chain performance and organizational performance: an empirical study in Malaysia. *Uncertain Supply Chain Management*.

[50] Alzoubi H., In M., Airat N., Ahmed G. (2022). Investigating the impact of total quality management practices and Six Sigma processes to enhance the quality and reduce the cost of quality: the case of Dubai. *International Journal of Business Excellence*.

[51] Hamadneh S., Pedersen O., Alshurideh M., Kurdi B., Alzoubi H. (2021). An investigation of the role of supply chain visibility into the scottish blood supply chain. *Journal of Legal, Ethical and Regulatory Issues*.

[52] Abdou H. A., Ellelly N. N., Elamer A. A., Hussainey K., Yazdifar H. (2021). Corporate governance and earnings management nexus: evidence from the UK and Egypt using neural networks. *International Journal of Finance & Economics*.

[53] Tang Z., De Almeida C., Fishwick P. (1991). Time series forecasting using neural networks vs. box-jenkins methodology. *Simulation*.

